# Identifying interactions between TDP‐43's N‐terminal and RNA‐binding domains

**DOI:** 10.1002/pro.70295

**Published:** 2025-09-17

**Authors:** David D. Scott, Lipsa Jena, Akash Rajaram, Jason Ang, Samantha Perez‐Miller, Vlad Kumirov, Rajesh Khanna, May Khanna

**Affiliations:** ^1^ Department of Pharmacology and Therapeutics, College of Medicine University of Florida Gainesville Florida USA; ^2^ Department of Chemistry and Biochemistry, College of Science University of Arizona Tucson Arizona USA

**Keywords:** CPMG NMR, HSQC‐NMR interdomain interaction, *MST*, TDP‐43

## Abstract

TAR DNA‐binding Protein 43 kilodaltons (TDP‐43) plays a crucial role in the pathophysiology and progression of amyotrophic lateral sclerosis, affecting familial and sporadic cases. TDP‐43 is an intrinsically disordered multidomain protein that consists of an N‐terminal domain (NTD_1–102_), two tandem RNA recognition motifs (RRM1_102–177_ and RRM2_191–260_), and an intrinsically disordered glycine‐rich C‐terminal_261–414_ domain. We previously identified a chemical probe that led to allosteric alterations between the RRM and NTD of TDP‐43. We attributed these changes to potential interdomain interactions between the NTD and RRM segments. In this work, we compared the 2D [^1^H,^15^N] HSQC‐NMR resonances of two constructs, TDP‐43_102–260_ (RRM domain alone) against TDP‐43_1–260_ (NTD linked to RRM) and observed clustered shifts in the RNA‐binding sites of both RRM domains. To investigate why these shifts appeared in the RRM domains in the absence of RNA, we hypothesized that the NTD domain could be stacking on the RRM domains. Thus, we modeled NTD–RRM interactions using protein–protein docking of TDP‐43 subdomains that propose NTD stacking onto the RRM domains. Using Carr‐Purcell‐Meiboom‐Gill NMR spectroscopy, we demonstrated evidence of an interaction between NTD_1–102_ and RRMs_102–260_. Finally, we investigated the impact of NTD on RNA binding using 2D ^15^N‐HSQC‐NMR and microscale thermophoresis by titration of a short UG‐rich RNA sequence and observed significant changes in RNA binding between TDP‐43_102–260_ and TDP‐43_1–260_, further suggesting the NTD plays a role in TDP‐43 RNA interactions.

## 1 INTRODUCTION

Transactive Response (TAR) DNA‐binding Protein 43 kilodaltons (TDP‐43) is a ubiquitously expressed heterogeneous ribonucleoprotein (hnRNP) implicated in the pathophysiology of amyotrophic lateral sclerosis (ALS) and frontotemporal dementia (FTD), in addition to limbic‐predominant age‐related TDP‐43 encephalopathy (LATE) Alzheimer's disease (AD) (Arai et al., 2006; Kabashi et al., 2008; Neumann et al., 2006; Sreedharan et al., 2008). The likely underlying etiology encompasses a homeostatic imbalance between nuclear and cytoplasmic localization (Barmada et al., 2010), formation of aberrant inclusions composed of ubiquitinated and hyper‐phosphorylated TDP‐43 (Barmada et al., 2010; François‐Moutal, Perez‐Miller, et al., 2019; Prasad et al., 2019), and an increase in proteolytic cleavage of cytoplasmic TDP‐43 (Kumar et al., 2023). TDP‐43 consists of an N‐terminal domain (NTD), two tandem RNA recognition motifs (RRM), RRM1 and RRM2, and an intrinsically disordered prion‐like C‐terminal domain (CTD) (François‐Moutal, Perez‐Miller, et al., 2019). It was demonstrated that the NTD and RRM domains contribute to TDP‐43 pathology and possibly amyloid formation (Agrawal et al., 2019; Garnier et al., 2017; Weskamp et al., 2020), highlighting the need to fully understand the structural role of these domains in TDP‐43 pathology.

Our laboratory, driven by the potential of TDP‐43 as a target for treating neurodegenerative diseases, has focused on identifying druggable sites on TDP‐43, particularly those involved in RNA/protein interactions. We identified compounds targeting the RNA‐binding domain yielding rTRD01 (François‐Moutal, Felemban, et al., 2019) and the NTD of TDP‐43 yielding nTRD22 (Mollasalehi et al., 2020). While developing these chemical probes, we discovered potential allosteric modulation of RNA binding. nTRD22, while specifically targeting the NTD of TDP‐43 and not binding to the RRM domains, causes changes in the NMR spectrum peaks of the RRM domains and inhibits typical RNA binding, suggesting an allosteric modulation (Hawkins & Lamb, 1995; Mollasalehi et al., 2020). Given these observations, we hypothesized that the NTD and RRM domains might communicate with each other either through allostery or direct interaction, as previously proposed (Wei et al., 2016). This was supported by a recent small‐angle x‐ray scattering (SAXS) study showing NTD interacting with the RRM domains. However, the TDP‐43 construct in this study had all tryptophan residues mutated to alanine residues (Wright et al., 2020).

In this study, we proposed the hypothesis that the NTD might interact closely with the RRM domain, and this interaction could play a crucial role in RNA binding, especially in the case of weaker, non‐specific RNA binding. We (i) reveal, via NMR, the shift of residues in the RRM domain when the NTD is present, suggesting that the NTD is interacting with the RRM domain, (ii) employ molecular modeling to validate this as a plausible conformation, and (iii) demonstrate that RNA exhibits different binding modes depending on whether the NTD is present or absent.

## 2 METHODS

### 2.1 Materials

Unless otherwise indicated, all reagents were purchased from Sigma (St. Louis, MO, USA), Fisher Scientific (Hampton, NH), and IDT DNA.

### 2.2 Purification of ^15^N‐labeled recombinant TDP‐43 subdomains

Human TDP‐43_102–260_ and TDP‐43_1–260_ were expressed in *Escherichia coli* BL21(DE3) cells (Novagen) in luria broth (LB)‐rich or M9 minimal media supplemented with ^15^NH_4_Cl. After the OD_600_ reached 0.8, 0.5 mM isopropyl β‐D‐1‐galactopyranoside was used to induce protein expression at 20°C for 24 h. Cells were collected by 4500 rpm centrifugation and resuspended in 40 mM HEPES, pH 7.5, 500 mM NaCl, 5 mM dithiothreitol (DTT), 30 mM imidazole, and ethylenediaminetetraacetic acid (EDTA)‐free protease inhibitor cocktail. Cell disruption was performed by three rounds of high‐pressure homogenization at 15,000 PSI with an LM10 Microfluidizer (Microfluidics, Westwood) and cell debris was removed by centrifugation at 50,000 rpm for 1 h at 4°C. The supernatant was then loaded on a His‐Trap (GE Healthcare, Uppsala, Sweden) previously equilibrated using the lysis buffer. The protein was then eluted with a linear gradient of imidazole up to 400 mM. The fractions of interest were loaded on a HiLoad Superdex size exclusion column (GE Healthcare, Uppsala, Sweden) and eluted with 20 mM HEPES pH 6.5, 300 mM NaCl, 10 mM DTT. Protein was concentrated with Amicon Ultra 15 centrifugal filters (regenerated cellulose 10,000 NMWL; Merck Millipore, Darmstadt, Germany) and concentration was determined by a Pierce assay using bovine serum albumin as a standard. When needed, protein aliquots were pooled, and Tobacco Etch Virus was added at a 1:100 ratio and left overnight at 4°C. Cleaved protein was recovered by loading onto a His‐Trap column and collecting the flow‐through. The purity of the protein was verified with sodium dodecyl sulfate–polyacrylamide gel electrophoresis. The proteins were flash frozen in liquid nitrogen and stored at −80°C.

### 2.3 Purification of recombinant TDP‐43 N‐terminal domain

Human TDP‐43_1–102_ was expressed in *E. coli* BL21(DE3) cells (Novagen) in LB‐rich or M9 minimal media supplemented with ^15^NH_4_Cl. After the OD_600_ reached 0.8, 1 mM isopropyl β‐D‐1‐galactopyranoside was used to induce protein expression at 30°C overnight. Purification was performed identically to the other TDP‐43 subdomain constructs and stored in the NMR buffer. Human TDP‐43_1–77_ was expressed in *E. coli* BL21(DE3) cells (Novagen) in LB‐rich media. After the OD_600_ reached 0.8, 1 mM isopropyl β‐D‐1‐galactopyranoside was used to induce protein expression at 20°C overnight. Purification and storage were identical to TDP‐43_1–102_.

### 2.4 2D ^15^N‐HSQC‐NMR

All NMR data were collected in NMR buffer: 40 mM HEPES pH 6.5, 300 mM NaCl, and 4 mM DTT on a Bruker Avance NEO 800 MHz spectrometer with TCI‐H&F/C/N probe at 25°C. A transverse relaxation optimized spectroscopy (TROSY) (Takeuchi et al., 2015) with a solvent suppression pulse sequence was used to acquire heteronuclear single‐quantum coherence‐ nuclear magnetic resonance (HSQC) data for all ^15^N labeled HisTDP‐43 at 25 μM. Under the same conditions, (UG)_4_ was titrated at a 1:2 molar ratio for all constructs. NMR data processing and analysis were performed using the programs NMRPipe (Delaglio et al., 1995) and Sparky (Lee et al., 2015). Chemical shift differences were calculated using the following equation (Williamson, 2013):

$$d=\sqrt{\frac{1}{2}\left[\delta H^{2}+\left(0.14\delta N^{2}\right)\right]}.$$

Chemical shifts greater than three standard deviations (*σ*) were considered significant.

### 2.5 Homologous time‐resolved fluorimetry of hisTDP‐43 and biotin (UG)_4_

His TDP‐43 and biotinylated (UG)_4_ were diluted to 4× working concentration in the assay buffer (40 mM HEPES pH 6.5, 300 mM NaCl, 4 mM DTT, 0.1% bovine serum albumin, and 0.05% Tween‐20). A 15‐point serial dilution of b(UG)_4_ was performed in the assay buffer. Five microliters of his TDP‐43 and b(UG)_4_ were mixed in a 384‐well plate and incubated for 30 min at room temperature. LANCE Eu‐W1024 Anti‐6xHis (AD0400, Perkin Elmer) and Streptavidin‐GL665 (610SAXLF, Cisbio) were diluted to a 2× working concentration in the assay buffer. Ten microliters of antibody and streptavidin mixture were added to the 384‐well plate wells and incubated for 1 h at room temperature. Fluorescence was collected using a CLARIOstar plate reader (BMGLabtech) at 620 and 665 nm.

### 2.6 In silico docking using ClusPro

The ClusPro server was used for protein–protein docking (Kozakov et al., 2013; Kozakov et al., 2017; Vajda et al., 2017). Briefly, ClusPro performs exhaustive sampling of ligand positions on the target and then scores by clustering the lowest‐energy structures using four different scoring functions: (1) balanced, (2) electrostatic‐favored, (3) hydrophobic‐favored, and (4) van der Waals plus electrostatic‐favored. The ligand was the first NMR model of the NTD (Protein Data Bank (PDB): 2N4P; Mompeán et al., 2016), prepared by removing the 6‐His‐tag. The target was the first model of the RRM1‐RRM2‐RNA NMR structure (PDB: 4BS2) (Lukavsky et al., 2013), with the 6‐His‐tag, the C‐terminal 10 amino acids linker, and the RNA molecule clipped to (UG)_4_ before docking. Default parameters were used. Docking was repeated using attractors on RRM2 based on chemical shift perturbation (CSP) shifts, but the results were nearly identical to the runs without attractors and are thus not shown. However, obtaining similar models using different parameters lends confidence to the output (Vajda et al., 2017).

### 2.7 Molecular modeling using high ambiguity drive protein–protein docking

Structures of the NTD (PDB: 5MRG; Mompeán et al., 2017) and RRMs (PDB 4BS2 (Lukavsky et al., 2013)) were simulated as protein–protein interactions using high ambiguity drive protein–protein docking (HADDOCK). It uses ambiguous interactions restraints (AIRs) from experimental data in combination with three‐step simulated interaction using rigid‐body docking, semiflexible refinement, and minimized by molecular dynamics simulations in an explicit solvent shell. Using default settings, TDP‐43 structures along with predefined “active” residues from the CSP analysis were submitted to the HADDOCKv2.4 web server. HADDOCK assembled 92 structures into 13 clusters, representing 46% of all water‐refined models generated. The results were ranked using the standard energy scoring metric in HADDOCK and three Clusters, #2, #3, and #5 containing 25 structures showed favorable HADDOCK scores with negative *Z*‐score values (Table 1).

**TABLE 1:** Results of top three clusters from high ambiguity drive protein–protein docking (HADDOCK) protein–protein docking simulations of TAR DNA‐binding Protein 43 kilodaltons (TDP‐43_1–102_) and TDP‐43_102–260_.

HADDOCK results	HADDOCK cluster 2	HADDOCK cluster 3	HADDOCK cluster 5
HADDOCK score	−83.5 ± 3.3	−82.0 ± 11.2	−75.7 ± 7.7
Cluster size	9	8	7
RMSD from the overall lowest‐energy structure	7.3 ± 0.1	0.9 ± 0.5	5.4 ± 0.3
Van der Waals energy	−61.0 ± 7.6	−66.2 ± 1.2	−54.5 ± 6.1
Electrostatic energy	−221.3 ± 22.8	−262.1 ± 63.5	−242.7 ± 45.7
Desolvation energy	16.7 ± 1.4	27.2 ± 2.9	24.2 ± 2.3
Restraints violation energy	51.1 ± 37.9	93.9 ± 35.6	30.5 ± 30.2
Buried surface area	1691.5 ± 31.2	1921.3 ± 34.4	1585.3 ± 125.2
*Z*‐score	−1.6	−1.4	−0.8

### 2.8 Microscale thermophoresis

The UG repeats (UG)_4_: rUGrUGrUGrUG and (UG)_6_: rUGrUGrUGrUGrUGrUG were purchased from Integrated DNA Technologies, Inc (IDT DNA). The UG repeats ((UG)_*n*_) were resuspended in PBS‐0.05% T buffer provided by Monolith His‐Tag Labeling Kit RED‐tris‐NTA 2nd generation (Nanotemper, Germany) to stock concentrations of 5 mM and stored at −20°C.

HisTDP‐43_1–260_, HisTDP‐43_102–260_, and the UG repeats were diluted to their working concentrations in the assay buffer (Monolith's PBS‐0.05%T). The protein was labeled according to the instruction manual provided by the manufacturer (Nanotemper, Germany). Briefly, 200 nM of the protein was labeled with 100 nM NT‐647 dye of Monolith His‐Tag Labeling Kit RED‐tris‐NTA 2nd Generation (Nanotemper, Germany) and incubated at room temperature for 30 min, followed by centrifugation at 15,000 × g for 10 min at 4°C. A 16‐point serial dilution of each UG repeat was prepared for the assay. Ten microliters of each of labeled HisTDP‐43 and (UG)_*n*_ were mixed in a tube at different concentrations of ((UG)_*n*_). The final concentration of the labeled HisTDP‐43 in the assay was 50 nM. For assays with labeled HisTDP‐43_1–260_, thermophoresis signals were captured using microscale thermophoresis (MST) premium capillaries at 60% excitation power and 40% MST power on a Monolith NT.115 device (Nanotemper, Germany). The data were analyzed with MO Affinity Analysis software (Nanotemper), utilizing the Hill model, and fitted using the specific binding with the Hill slope model in GraphPad Prism 9.1.2. For assays with labeled HisTDP‐43_102–260_, the thermophoresis signals were captured at 650 and 670 nm using MST premium capillaries at 80% excitation power and medium MST power on a Monolith X device (Nanotemper, Germany). The data, analyzed as a ratio of 670/650 nm, were fitted using the Kd model for (UG)_6_ and the Non‐Binder model for (UG)_4_ in MO Control 2 software and then plotted in GraphPad Prism 10.2.3.

### 2.9 Native protein gel shift

Native protein gel electrophoresis was performed using a 4%–16% Bis‐Tris polyacrylamide gel (Invitrogen) under non‐denaturing conditions. Samples containing NTD (TDP‐43_1−77_) and RRM (TDP‐43_102–260_) were prepared at varying concentrations, including individual domains and mixtures at molar ratios of 0.5:1, 1:1, and 2:1 (NTD:RRM). Prior to electrophoresis, samples were incubated at 25°C for 10 min in buffer containing 300 mM NaCl, 40 mM 4‐(2‐hydroxyethyl)‐1‐piperazineethanesulfonic acid (HEPES) (pH 6.5), and 4 mM DTT. Following incubation, samples were mixed with native loading buffer, loaded onto the gel, and ran at 150 V for 90 min. Protein bands were visualized by Coomassie Brilliant Blue (BioRad) staining. For RNA‐containing samples, RNA was added after the initial 10‐min protein incubation, followed by an additional 20‐min incubation at the same temperature.

### 2.10 Native RNA gel shift

Electrophoretic mobility shift assay (EMSA) was conducted using a non‐denaturing 0.5× tris‐borate‐EDTA (TBE) 6.25% polyacrylamide gel in 0.5× TBE running buffer. The gel was pre‐run at 100 V for 60 min at 4°C. One micromolar of TDP‐43_102–260_ and various concentrations of TDP‐43_1–77_ were first incubated at 25°C for 10 min in 300 mM NaCl, 40 mM HEPES pH 6.5, and 4 mM DTT. After the addition of (UG)_6_ RNA, the samples were incubated at the same temperature for another 20 min. Samples were then mixed with non‐denaturing loading buffer, loaded into the gel, and run at 100 V for 60 min at 4°C. RNA bands were visualized using SYBR Gold staining.

## 3 RESULTS

### 3.1 2D [^1^H,^15^N] HSQC‐NMR of TDP‐43_102–260_ versus TDP‐43_1–260_

**FIGURE 1:**
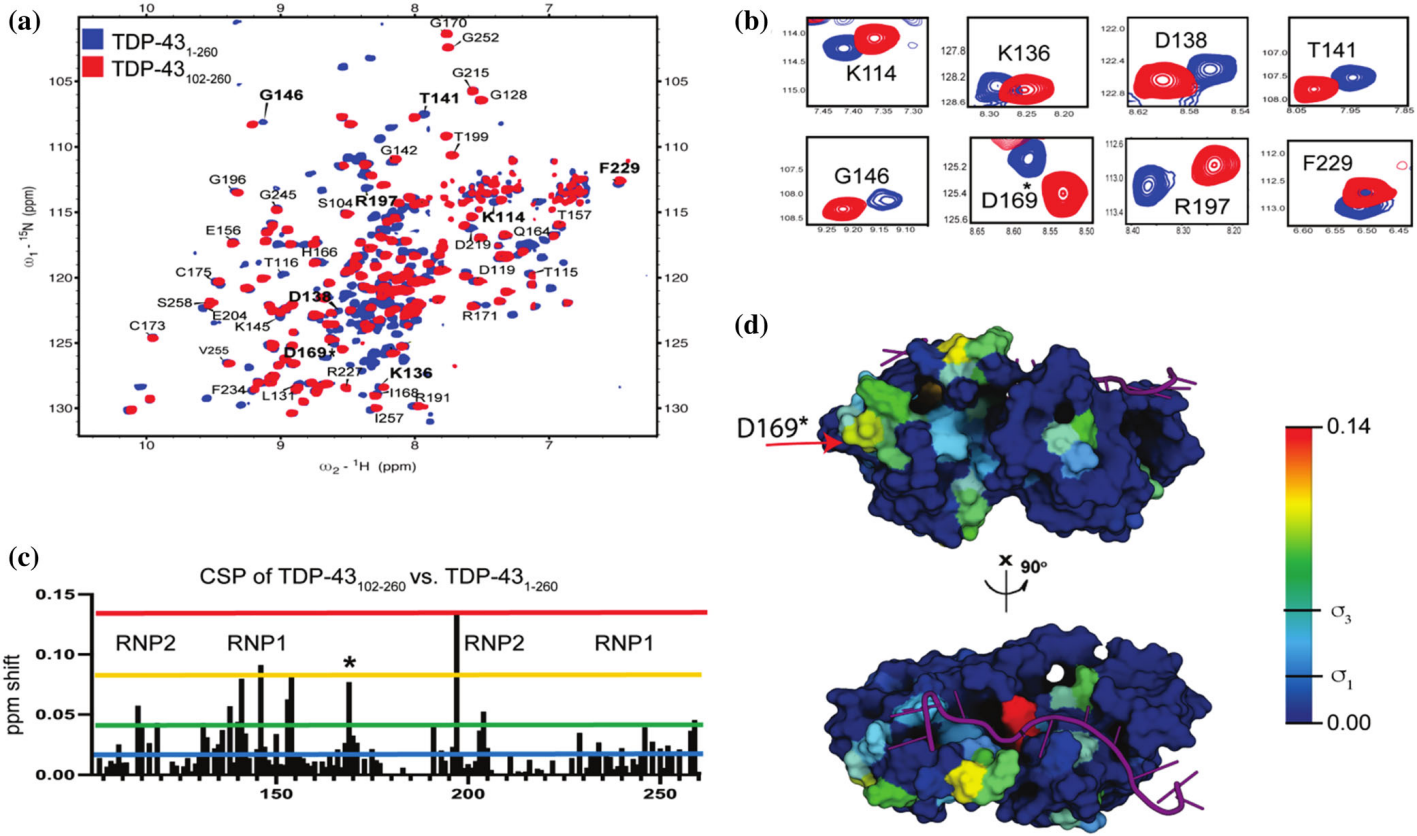
2D HSQC‐NMR of TAR DNA‐binding Protein 43 kilodaltons (TDP‐43_1–260_) identifies N‐terminal domain–RNA recognition motifs (NTD–RRM) interactions (a) 2D ^15^N‐HSQC‐NMR overlay of ^15^N and ^1^H resonances of TDP‐43_102–260_ (red) onto ^15^N TDP‐43_1–260_ (blue) at 25 μM under identical conditions. (b) Zoomed regions of 2D HSQC‐NMR overlay show exemplar polar residues of the RRM domains that are shifted in the presence of the NTD. (c) Chemical shift plot (CSP) from resonances of TDP‐43_102–260_ vs. TDP‐43_1–260_ shows shifts in the RNP‐1 and RNP‐2. RNP‐1 and RNP‐2 are short motifs in the RRM domains known as ribonucleoprotein domains (Maris et al., 2005). (d) CSP analysis was projected as a heat map representative of shift intensities onto the tandem RRM RNA‐bound structure (PDB: 4BS2) (Sun et al., 2021). Colored lines represent projected values of heatmap, with the blue and green bar being the standard deviation and 3× standard deviation, respectively (0.014, 0.041).

We examined the effect of NTD on RRM domains by comparing the 2D [^1^H,^15^N] HSQC‐NMR spectra of two TDP‐43 constructs, one containing the RRM domain with NTD and one without NTD (Figure 1). For the remainder of the manuscript, we refer to these constructs as follows: TDP‐43, which covers only the RRM domains (RRM1 and RRM2) from 102 to 260 amino acids, is designated TDP‐43_102–260_; the construct that covers the NTD and the RRM domain will be TDP‐43_1–260_ and covers amino acids 1–260 and the construct that covers only the NTD 1–102 as TDP‐43_1–102_ (Figure S1, Table 2). TDP‐43_102–260_ spectra reproducibly display a well‐folded protein indicated by the sharp, well‐resolved peaks as seen before with 143 identified resonances (Scott et al., 2019) (Figures 1 and S3A). TDP‐43_1–260_ contains three folded domains: NTD, RRM1, and RRM2. Based on this, we expect ~222 amide peaks in the ^15^N‐HSQC‐NMR acquired spectra. For the first time, we show a high‐resolution 2D [^1^H,^15^N] TROSY‐HSQC spectrum of multiple TDP‐43 domains connected in tandem by acquiring TDP‐43_1–260_ in low protein concentrations, resulting in 208 combined resonances from the NTD and RRMs (Figure S3A–C). Superimposing the TDP‐43_102–260_ NMR spectrum onto TDP‐43_1–260_ results in overlapping peaks from the RRM region and distinguishable peaks from NTD resonances (Figure 1a). By transferring ^1^H,^15^N assigned resonances of apoTDP‐43 RRM domains to the superimposed spectra, we found significant chemical shifts primarily of charged residues in the RRM domains (Figure 1b). CSP analysis shows significant clustered shifts in the ribonucleoprotein sequences of RRM1 and RRM2 (Figure 1c). There are at least three clusters of changes that include a few residues in the NTD, residues RRM1 (RNP1) and residues in the RRM2 (RNP2) regions. Next, we mapped the chemical shifts onto the RNA‐bound tandem RRM structure, revealing critical residues involved in RNA binding that are appreciably perturbed by the presence of the NTD in TDP‐43_1–260_ (Figure 1d). Of importance to ALS and mutations found in TDP‐43 connected to ALS, one of the most significantly shifted residues is D169, which is a mutated to a glycine residue in ALS patients (Figure 1b,d), previously reported to (i) disrupt the ATP‐binding capacity of the RRM1 domain (Dang & Song, 2020), (ii) disrupt phase separation‐induced by NEAT1 RNA binding to TDP‐43 (Wang et al., 2020), and (iii) was shown to increase thermal stability of a TDP‐43(1–265) construct (Chiang et al., 2016).

**TABLE 2:** TAR DNA‐binding Protein 43 kilodaltons (TDP‐43_1–77_) constructs, molecular weight (MW), and isoelectric point.

Construct	No.of residues	MW	pI	pI Khanna tag	pI Genscript tag	Sequence
TDP43_1–77_	77	8.52	4.3	5.83	5.28	MSEYIRVTEDENDEPIEIPSEDDGTVLLSTVTAQFPGACGLRYRNPVSQCMRGVRLVEGILHAPDAGWGNLVYVVNY
TDP43_1–102_	102	11.31	5.1	6.7	6.03	MSEYIRVTEDENDEPIEIPSEDDGTVLLSTVTAQFPGACGLRYRNPVSQCMRGVRLVEGILHAPDAGWGNLVYVVNYPKDNKRKMDETDASSAVKVKRAVQK
TDP43_102–260_	158	18.31	5.65	6.79	6.19	KTSDLIVLGLPWKTTEQDLKEYFSTFGEVLMVQVKKDLKTGHSKGFGFVRFTEYETQVKVMSQRHMIDGRWCDCKLPNSKQSQDEPLRSRKVFVGRCTEDMTEDELREFFSQYGDVMDVFIPKPFRAFAFVTFADDQIAQSLCGEDLIIKGISVHISNA
TDP43_1–260_	260	29.5	5.27	6.13	5.78	MSEYIRVTEDENDEPIEIPSEDDGTVLLSTVTAQFPGACGLRYRNPVSQCMRGVRLVEGILHAPDAGWGNLVYVVNYPKDNKRKMDETDASSAVKVKRAVQKTSDLIVLGLPWKTTEQDLKEYFSTFGEVLMVQVKKDLKTGHSKGFGFVRFTEYETQVKVMSQRHMIDGRWCDCKLPNSKQSQDEPLRSRKVFVGRCTEDMTEDELREFFSQYGDVMDVFIPKPFRAFAFVTFADDQIAQSLCGEDLIIKGISVHISNA
His‐tag Genscript	21	2.42				MGSSHHHHHHSSGENLYFQGH
His‐tag Khanna	34	3.56				MGSSHHHHHHSSGLVPRGSHMASMTGGQQMGRGS

### 3.2 Prediction of interdomain contacts by simulating protein–protein interactions

To further understand how the NTD interacts with RRM domains, structures of the NTD (PDB: 5MRG; Mompeán et al., 2017) and RRMs (PDB: 4BS2; Lukavsky et al., 2013) were simulated as protein–protein interactions using HADDOCK (de Vries et al., 2010; van Zundert et al., 2016).

**FIGURE 2:**
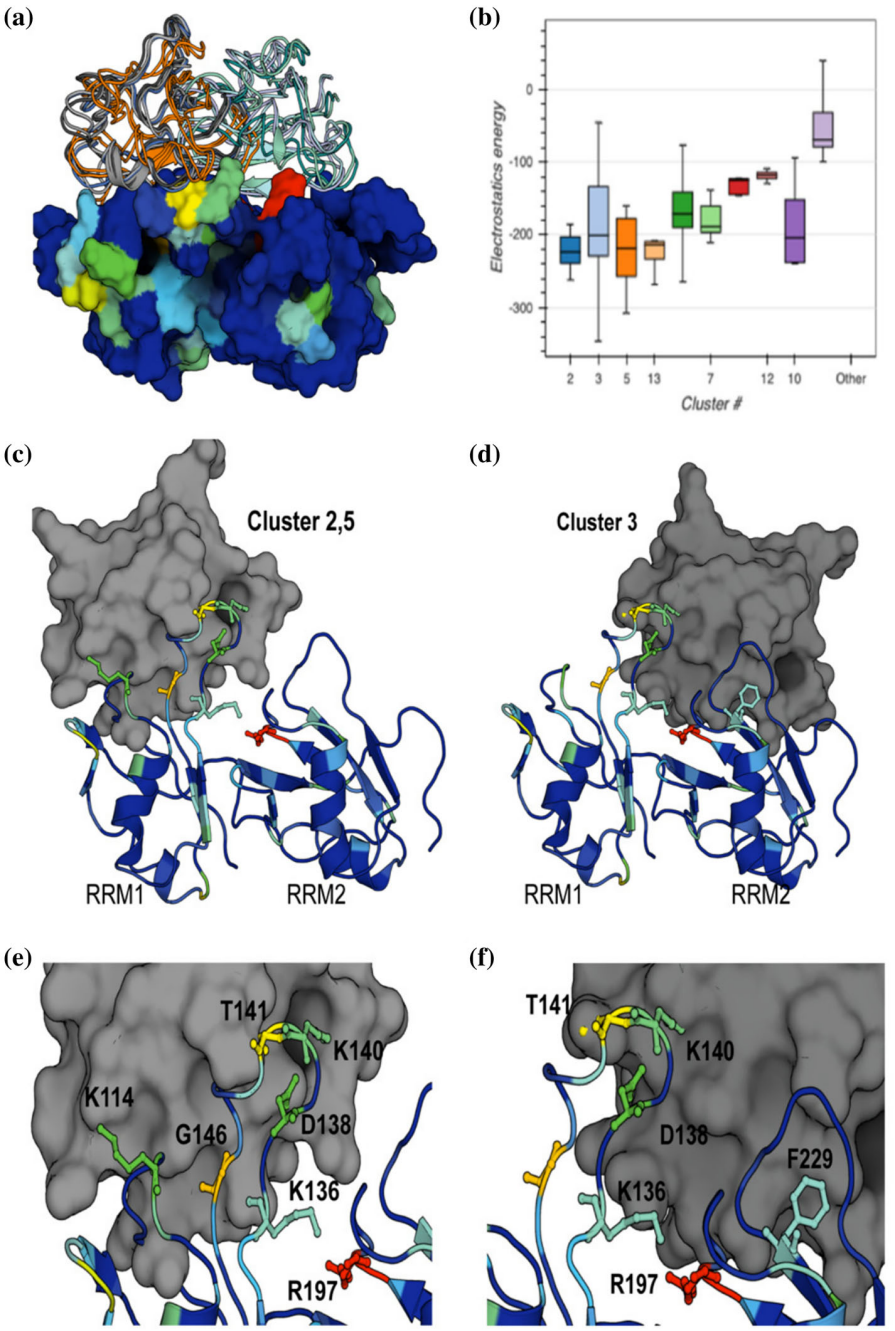
High ambiguity drive protein–protein docking (HADDOCK) protein–protein simulations of N‐terminal domain–RNA recognition motifs (NTD–RRM) stacking. (a) Top 3 HADDOCK clusters contain 25 structures stacking the NTD (cartoons) onto the RRM domains (surface). The chemical shift plot (CSP) heat map from 2D HSQC‐NMR is used to show correlations between experimental and simulated data. (b) HADDOCK results were ranked by HADDOCK score, *Z*‐score, RMSD, and electrostatic potential energy. A general trend was seen that the lower‐RMSD containing top clusters also had the lowest potential electrostatic energy. (c) HADDOCK Clusters 2 and 5 stacks the NTD (gray) onto RRM1. (d) Cluster 3 stacks NTD (gray) onto the RRM2 domain. (e) Zoom of Clusters 2 and 5 stacked onto RRM1 with significant shifts from CSP analysis labeled. (f) Zoom of Cluster 3 stacked onto RRM2 with significant shifts from CSP analysis labeled.

Visualization of these docking results predicts NTD interacting with both RRM domains but with a tendency toward RRM1 (Figure 2a). A notable pattern emerges when comparing clusters of HADDOCK score against root mean square deviation (RMSD) and total electrostatic energy, where the most favorable clusters exhibit the lowest electrostatic potential energy. This underscores the significance of electrostatic residues, as observed in the 2D ^15^N‐HSQC‐NMR, which becomes apparent with the addition of the NTD to the construct (Saponaro et al., 2020) (Figure 2b). Of these top clusters, #2 and #5 were analogous in values for HADDOCK score, RMSD, and electrostatic energy, with both results stacking the NTD onto RRM1, while cluster #3 shows the NTD stacking onto RRM2 (Figure 2c,d). From our CSP analysis, K136, D138, T141, and G146 of RRM1, along with R197 and F229 of RRM2, were the most significantly perturbed residues and most likely sites of NTD interactions. Using the heat map projected onto the RRM structure from our CSP analysis, visualization of all HADDOCK clusters correlated well with the predicted interactions of both RRM domains (Figure 2e,f). We repeated the NTD–RRM interactions using the ClusPro protein–protein webserver (Kozakov et al., 2017) using the same structures. ClusPro only uses rigid‐body docking with inter‐ and intramolecular interaction energies evaluated by force field calculations. ClusPro results also indicated NTD–RRM interdomain contacts, but all force field weights, including electrostatic, hydrophobic, or balanced, replicated NTD stacking onto RRM2 (Figure S4) (Desta et al., 2020; Vajda et al., 2017).

### 3.3 Biophysical assessment of NTD–RRM interactions using CPMG‐NMR

**FIGURE 3:**
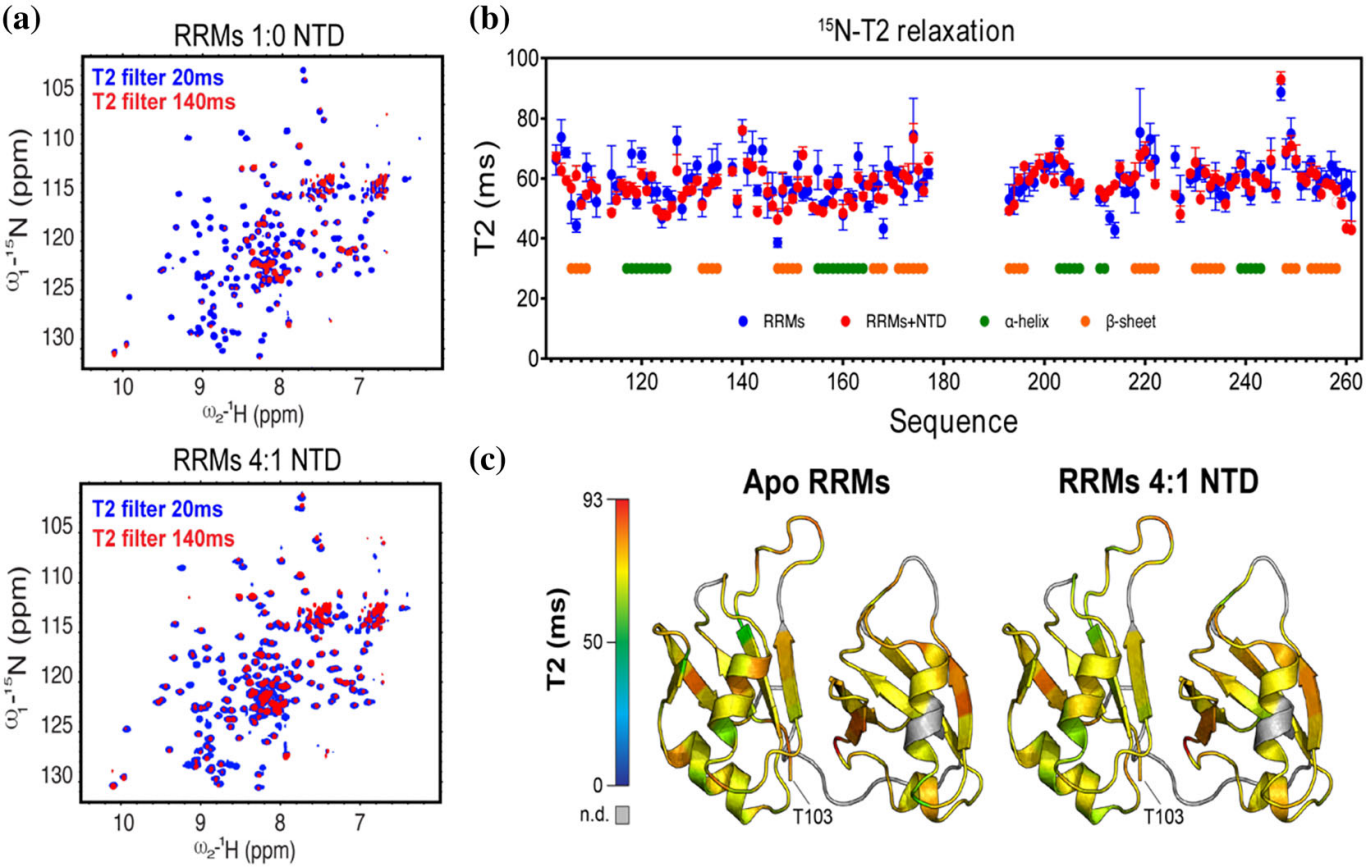
Carr‐Purcell‐Meiboom‐Gill NMR of protein–protein interactions between TAR DNA‐binding Protein 43 kilodaltons (TDP‐43_102–260_) and TDP‐43_1–102_. (a) CPMG NMR was acquired from ^15^N‐labeled TDP‐43_102–260_ at 100 μM with five T2 filters ranging from 20 to 540 ms. Twenty and 140 ms spectra are shown as representative of the decay rate for apoRRMs. Twenty‐five micromolar unlabeled NTD_1–102_ was added to ^15^N TDP‐43_102–260_ and the same T2 filters were acquired using CPMG NMR with five time points. Twenty and 140 ms spectra are shown as representative of the decay rate for apoRRMs. (b) Local T2‐relaxation constants were calculated for apo and bound TDP‐43_102–260_ at each T2 filter and (c) projected as a heat map onto the RRMs, showing a slight decrease in the loop region of RRM1. NTD, N‐terminal domain; RRM, RNA recognition motifs.

The transient formation of protein–protein interactions is challenging to characterize biophysically due to their dynamic nature and shallow interfaces. This complexity is further amplified by the differential behavior of TDP‐43 constructs across various pHs (Figure S2). NMR relaxation experiments excel in this area, as they can report time scales ranging from picoseconds to milliseconds (Theillet, 2022). As domain motion of large multidomain proteins occurs in the μs–ms timescale, Carr‐Purcell‐Meiboom‐Gill (CPMG) NMR spectroscopy is a valuable approach for measuring protein dynamics over a range of timescales (Dokainish & Sugita, 2020; Palmer 3rd, 2004). Using CPMG‐NMR, we quantified the line broadening that occurs by the chemical exchange between the ground state and one or more excited states of NMR resonances over specific time points (Gopalan et al., 2018; Hertel et al., 2019). 2D ^15^N‐HSQC‐NMR spectra of 100 μM TDP‐43_102–260_ were acquired with a CPMG filter of 20, 40, 70, 140, 270, and 540 ms. Among these, the 20, 70, and 140 ms displayed significant changes in signal intensity and are appropriate ranges for relaxation rates of 20 kDa proteins (Palmer 3rd, 2004). Using these same parameters, unlabeled NTD_1–102_ was added to ^15^N TDP‐43_102–260_ (RRM1 and RRM2) at a 1:4 (NTD:RRM1 and RRM2) molar ratio and showed significant changes in signal intensity compared to apoTDP‐43_102–260_ (Figure 3a). As the NTD readily oligomerizes, concentrations above a 1:4 molar ratio of NTD:RRM12 showed no signs of interaction and were likely necessary to compete with the oligomerization of NTD. Integration of each peak in the bound and unbound state provides a local relaxation constant used to compare relative dynamics indicative of transient binding modes. In doing so, we observed evidence of complex formation by a measured decrease in T2 relaxation of TDP‐43_102–260_ in solution with the NTD_1–102_ (Figure 3b,c) within the loop of RRM1 containing the RNP1 sequence.

### 3.4 NTD competes with RNA binding to the RRM domains in 2D ^15^N‐HSQC‐NMR and MST

**FIGURE 4:**
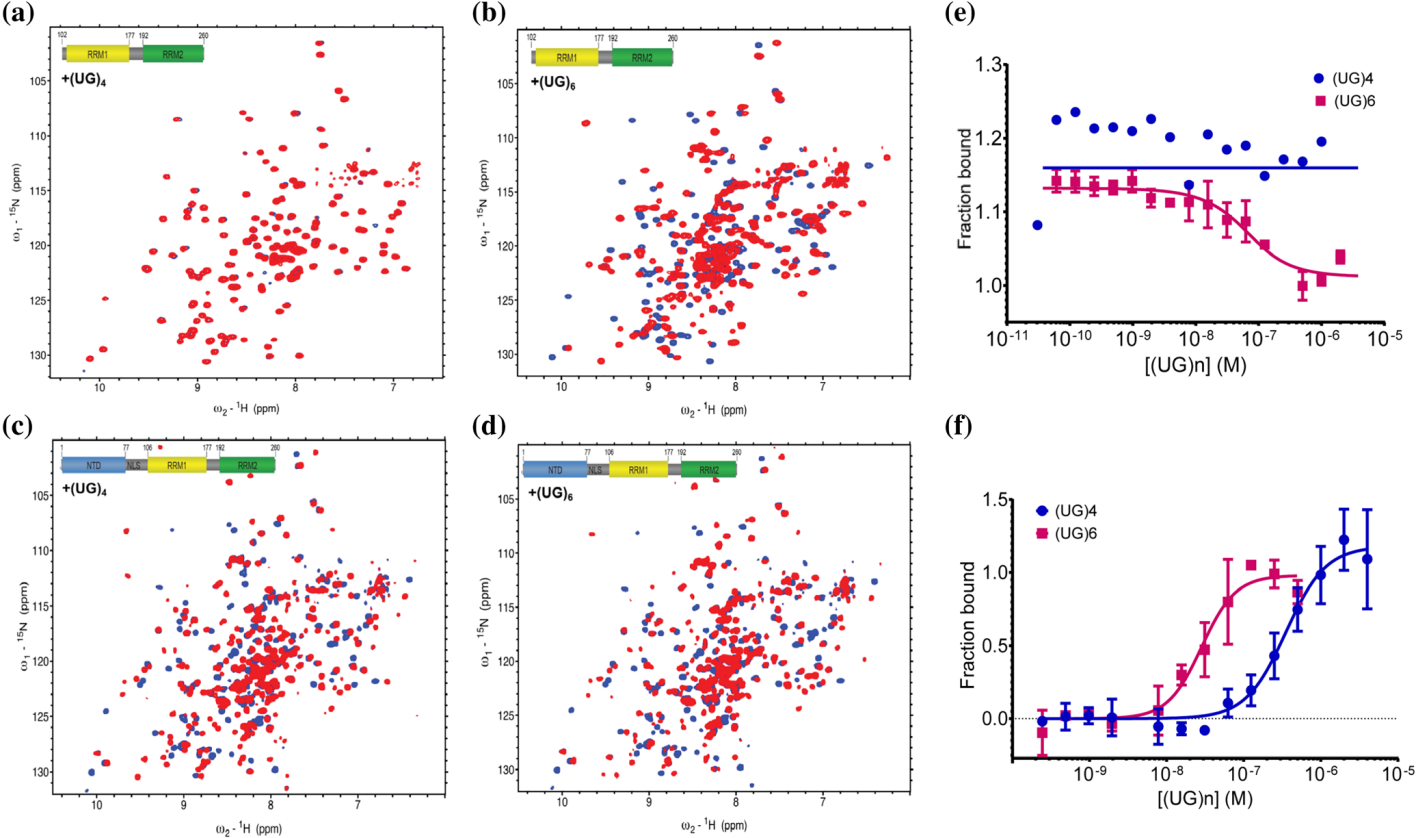
2D ^15^N‐HSQC‐NMR and microscale thermophoresis (MST) identifies a change in RNA recognition motifs (RRM)‐RNA‐binding dynamics in the presence of the N‐terminal domain. (a) 2D ^15^N‐HSQC‐NMR overlay of apoTDP‐43_102–260_ (blue) and (UG)_4_‐bound (red) at 1:1. (b) 2D ^15^N‐HSQC‐NMR overlay of apoTDP‐43_102–260_ and (UG)_6_‐bound (red) at 1:1. (c) 2D ^15^N‐HSQC‐NMR overlay of apoTDP‐43_1–260_ (blue) and (UG)_4_‐bound (red) at 1:1. (d) 2D ^15^N‐HSQC‐NMR overlay of apoTDP‐43_1–260_ and (UG)_6_‐bound (red) at 1:1. Significant chemical shifts are observed in RRM residues and N‐terminal domain (NTD) residues, indicating NTD plays a role in RNA binding to the RRM domains. (e) MST values taken at a 670 nm/650 nm ratio were used to determine the dissociation constant for (UG)_*n*_ binding to TDP‐43_102–260_. The data were fitted using MO Control 2 software as described in Section 2, resulting in an apparent Kd of 39.8 nM for (UG)_6_, with a confidence interval (CI) of 16.9–93.8 nM. For (UG)_4_, the MST values did not produce a binding curve or fit the Kd model. Instead, they fit the non‐binder model, indicating no binding interaction between TDP‐43_102–260_ and (UG)_4_. (f) MST values were used to determine the dissociation constant for binding of (UG)_*n*_ to TDP‐43_1–260_. The data were fitted as described in Section 2, yielding an apparent Kd of (UG)_4_ = 246.5 nM with a CI of 178.0–345.5 nM and that for (UG)_6_ = 28.42 nM with a CI of 21.28–37.7 nM. Data are represented by mean ± SD for *n* = 3 except for (UG)_4_ in panel (d) where *n* = 1 is represented.

Given that the NTD_1–102_ showed interaction with the RRM domains and was proposed to engage with crucial residues involved in RNA binding, we further explored the potential relationship between the interaction of NTD–RRMs and RNA binding. We identified specific differences between short RNA sequences binding to TDP‐43_102–260_ of the RRMs alone vs. TDP‐43_1–260_ that included the NTD. Because the TDP‐43 RRM domains exhibit a greater affinity for extended UG‐rich RNA repeats that promote cooperative RRM–RNA binding, we proposed using a shorter UG motif. We hypothesized that this motif would fall within optimal length of the RRM domain alone for 2D ^15^N‐HSQC‐NMR observation without increasing the number of interactions due to multiple TDP‐43 units and hence higher molecular weight formation that is problematic in NMR. However, titration of a short 5′‐UGUGUGUG‐3′ (henceforth referred to as: (UG)_4_) RNA onto TDP‐43_102–260_ and observation of changes using 2D ^15^N‐HSQC‐NMR resulted in no chemical shifts (Figure 4a). (UG)_4_ has two fewer nucleotides (eight) than the total number of RNA interaction sites of RRM1‐RRM2 (10). One possibility is that the 2D ^15^N‐HSQC‐NMR results show a fast exchange rate between (UG)_4_ and TDP‐43_102–260_, as is usually seen with low nM affinity ligands that generally go undetected by chemical shift analysis (Fielding, 2003; Srb et al., 2019; Theillet, 2022; Zartler & Shapiro, 2006). This spectrum is drastically different than the 2D ^15^N‐HSQC‐NMR spectrum of the canonical motif (UG)_6_, which displays significant chemical shifts indicative of slow‐exchange binding kinetics (Figure 4b).

Interestingly, we tested the same RNA sequences using 2D ^15^N‐HSQC‐NMR under identical conditions for TDP‐43_1–260_ and show significant chemical shifts for both UG4 and UG6—indicating that the presence of the NTD alters binding kinetics for RNA binding to the RRMs (Figure 4c,d). To further investigate this theory, we tested RNA binding to several TDP‐43 protein constructs using MST that allows the detection of interaction between a fluorescently labeled target (TDP‐43_102–260_ here) and a ligand ((UG)_*n*_) under an infrared laser that generates a temperature gradient in capillaries containing samples. MST resulted in no (UG)_4_ binding to TDP‐43_102–260_ (Figure 4e). Strikingly, titration of the same RNA onto TDP‐43_1–260_ shows a significant binding affinity with an apparent Kd of 246.5 nM (Figure 4f), indicative of significant conformational changes and consistent with the fast exchange observed by NMR (Figure 4b–d). To corroborate this further, we also tested the binding of a slightly longer UG motif (UG)_6_, which is four nucleotides longer than (UG)_4_ and two nucleotides longer than the total number of RNA interaction sites of RRM domains as a positive control for RNA‐binding event. We found that (UG)_6_ was successfully bound to both the TDP‐43 constructs with a 10‐fold increase in the binding affinity as compared to (UG)_4_ (Figure 4). These data suggest two possibilities: (1) in the absence of NTD, longer RNAs bind to the RRM domains covering the RNA interaction sites, and (2) shorter RNA binds to the RNA interaction sites only in the presence of NTD. To test this hypothesis, we assessed RNA binding in the presence and absence of the NTD using native gel electrophoresis.

### 3.5 Native gel of NTD binding to TDP‐43 RRM domains

**FIGURE 5:**
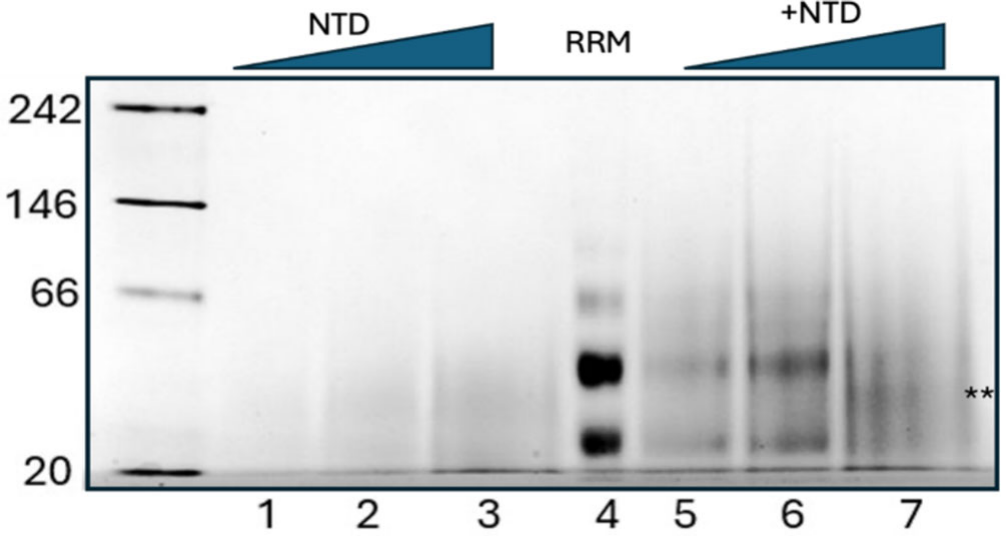
Native gel analysis of RNA recognition motifs (RRM) interaction with N‐terminal domain (NTD). A native gel illustrating the interaction between the RRM domain (TAR DNA‐binding Protein 43 kilodaltons [TDP‐43_102–260_]) and the NTD (TDP‐43_1–77_) of the protein. Lane contents are as follows: (1) 4.06 μM NTD, (2) 8.125 μM NTD, (3) 16.25 μM NTD, (4) 8.125 μM RRM alone, (5) 8.125 μM RRM + 4.06 μM NTD, (6) 8.125 μM RRM + 8.125 μM NTD, (7) 8.125 μM RRM + 16.25 μM NTD. Progressive shifts in the RRM band upon increasing concentrations of NTD indicate complex formation between the two domains.

We performed a native protein gel shift assay using the RRM domain of TDP‐43 (TDP‐43_102–260_) with only the structured portion of the NTD (TDP‐43_1–77_) (Figure 5). In the gel, there are two main forms observed for the RRM domain: monomer and dimer (Figure 5, Lane 4). The results show that the RRM domain undergoes a mobility shift upon the addition of NTD, indicating complex formation, although the shift is a smearing of the RRM upon the addition of the NTD (Figure 5, Lanes 5–7). Upon NTD addition, the monomer band shifts upward (Figure 5, Lane 7, starred), and the dimer (Figure 5, Lanes 5 and 6, top band) appears to shift down to this same position. A full shift, that is most readily observed by the disappearance of the monomer and dimer bands, is only observed at a 2:1 molar ratio of NTD to RRM, although it is more likely that in the native context, where NTD and RRM are covalently linked, the interaction may be more stable at a 1:1 ratio. Previously, we used a longer NTD construct and did not detect complex formation. We now suspect that the unstructured and highly basic loop between residues 77 and 102 may promote aggregation or interfere with complex formation by interacting with the structured portion of NTD. By isolating and focusing on the structured region alone, we likely stabilized an interaction that was previously undetectable. Notably, the shifted complex appears as a diffuse smear, rather than a sharp band, which may indicate phase separation or dynamic exchange within the complex. These observations further support the notion that the interaction may involve conformational flexibility or multivalent assembly. This is not surprising, since the NTD structure was previously solved and shown to form long multimers and can phase separate (Carter et al., 2021; Wang et al., 2018). We repeated the native gel electrophoresis experiment, this time staining for RNA rather than protein, to assess the impact of NTD on RRM:RNA interaction (Figures S7 and S8). In this gel, we assessed RNA binding of the RRM domain and observed a clear shift (Figure S7, Lane 3) upon the addition of TDP‐43_102–260_, indicating RNA binding. Notably, no RNA was detected in the RRM‐alone control (Figure S7, Lane 1). Upon the addition of increasing concentrations of the NTD (Figure S7, Lanes 4–9), we did not observe any disruption of RNA binding, which is consistent with the conclusion that the NTD does not interfere with specific RRM–RNA interactions. This experiment was repeated using a higher concentration of RNA and a larger range of NTD concentrations (Figure S8). Results were similar to those found in Figure S5. We conducted an additional native protein gel electrophoresis experiment, staining for protein, to assess the impact of RNA on NTD:RRM interaction (Figure S7). We observed that the NTD band gradually diminished with increasing RNA concentration, suggesting that the NTD is being sequestered into a higher‐order complex (Figure S9). This observation implies that the NTD may stabilize the RRM–RNA interaction under certain conditions, rather than disrupt it. Furthermore, given the high‐affinity nature of the TDP‐43/RNA interaction (in the nM–pM range), it is unlikely that the relatively weak μM‐range interaction between the NTD and RRM would disrupt RNA binding, that has been proposed to be as low as pM. Together, these results support the conclusion that the NTD does not compete with high‐affinity RNA for RRM binding and may, in fact, enhance complex stability in some contexts.

## 4 CONCLUSION

The work presented here elucidates the interaction between the NTD of TDP‐43 and its RRM domains. When comparing the RRM domain in a construct where the NTD is present, the 2D ^15^N‐HSQC resembles an RNA‐bound form. This suggests that stacking of the NTD onto the RRM domain affects the identical residues that would typically engage in RNA binding. This finding implies that the NTD may protect the RRM domain from non‐specific RNA binding.

To delve deeper into the finding that suggests an interaction between the NTD and the RNA‐binding residues of the RRM domains, we examined the impact of the NTD on the RRM and RNA binding through methods such as modeling, NMR spectroscopy, and MST. We acquired 2D [^1^H,^15^N] HSQC‐NMR spectra of tandem RRM domains TDP‐43_102–260_ and TDP‐43_1–260_, a construct containing the RRMs and NTD connected by TDP‐43's nuclear localization sequence. Superimposing the HSQC‐NMR spectrum of TDP‐43_102–260_ onto TDP‐43_1–260_ revealed significant chemical shifts within the RNA binding sites of the RRM domains.

To further explore the N‐terminal stacking onto the RRM domain, we modeled the NTD_1–102_ (PDB: 5MRG) and apo RRM's amino acids 102–260 (modified PDB: 4BS2) protein–protein interactions using High Ambiguity Driven protein–protein Docking (HADDOCK) and the ClusPro protein–protein docking server. These simulations showed NTD stacking onto RRM residues with high similarity to the shifted residues observed in NMR. Based on the modeling result, it is unclear if NTD stacks on RRM1 or RRM2 or overlaps these two domains. Our CSP analysis from the NMR data comparing the NTD‐containing construct to the RRM constructs seems to imply that it is skewed toward the RRM1 domain, with slight shifts of RRM2. However, the shifted residues were not assigned using multi‐dimensional NMR experiments, which could lead to errors of precisely which residues were most significantly shifted.

Commonly, titration experiments would be done using 2D ^15^N‐HSQC with varying ratios of the two proposed interacting domains, but because the NTD readily oligomerizes and aggregates, protein concentrations sufficient for titration were not possible. Therefore, we used CPMG‐NMR that measures the tumbling rate of protein in solution to verify the specific interaction between these two domains beyond their tandem connection. We observed a decreased T2 relaxation of TDP‐43_102–260_ when NTD_1–102_ was present, indicating higher molecular weight and complex formation, which further supports the interaction between the NTD and the RRM domain.

Considering the substantial therapeutic potential in targeting TDP‐43 and its pivotal role in the pathophysiology of numerous neurodegenerative diseases, it is crucial to understand how the apo structure of TDP‐43 diverges from the RNA‐bound structure and how the NTD domain can alter TDP‐43 function. With the new data underscoring the significance of the NTD's interaction with the RRM domain, we are led to question whether the NTD could impact RNA binding.

We examined the potential influence of the NTD on RNA binding. We hypothesized three possible outcomes of the NTD stacking on the RRM domain: (i) it could inhibit RNA binding, (ii) it could prevent weaker RNA binding without affecting canonical UG‐rich RNA binding, or (iii) it could enhance the binding of UG‐rich RNA. We tested a short RNA (eight nucleotides, (UG)_4_) and found that it binds differently to the RRM domain and the NTD–RRM construct. NMR data showed that (UG)_4_ binds strongly to the RRM domains, and although MST failed to measure the binding affinity, we were able to measure this interaction using homologous time‐resolved fluorimetry (HTRF) (Figure S6). Interestingly, the NTD–RRM construct (TDP‐43_1–260_) showed chemical shifts by NMR, indicating a lower binding affinity for short RNA in the presence of the NTD. In contrast, (UG)_6_ binds to both constructs with nearly identical chemical shifts and measured affinity by MST. This suggests that the NTD does not inhibit RNA binding, as (UG)_6_ binds to NTD–RRM in the same way it does to the RRM domain. Based on our current data, we cannot determine whether the NTD enhances the binding of the (UG)_4_ RNA or if the increased affinity is due to residues in the loop region that connect the NTD to the RRM domain (residues 77–102), which are heavily charged. This implies that the RRM alone has sufficient affinity to bind to short RNAs, with an increase in charge either from the loop region on the NTD or possibly from charged residues on the C‐terminal region of TDP‐43 not included in our studies (nine additional amino acids are included in the original structures of TDP‐43102‐269 with RNA; Lukavsky et al., 2013).

Our study highlights how stacking the TDP‐43 NTD covers the RRM domains through highly charged interactions, altering the RNA‐binding affinities and specificity. We show that there are different outcomes to binding small RNAs when the NTD stacks on the RRM. This may be important to regulate or prevent non‐specific interactions of TDP‐43 with RNAs of low affinity and length. This implicates a significant impact on studies that use tagged NTD constructs, which may disrupt this protective stacking and shift the protein toward a more pathological form of TDP‐43. In turn, this may drive aggregation otherwise prevented by constitutive NTD stacking. Indeed, one interpretation of overexpressed tagged TDP‐43 and its predilection for aggregation could be due to non‐specific RNA and possibly charged protein binding in the absence of NTD stacking. Future studies should thus focus on understanding if and how the NTD protects against aggregation while moving forward with a novel caveat for studies that use N‐terminal tagged TDP‐43.

## AUTHOR CONTRIBUTIONS

**David D. Scott:** Conceptualization; investigation; methodology; validation; writing – review and editing; visualization; writing – original draft. **Lipsa Jena:** Methodology; validation; writing – review and editing; visualization; investigation. **Akash Rajaram:** Methodology; validation; writing – review and editing; visualization; investigation. **Jason Ang:** Methodology; validation; writing – review and editing; visualization; investigation. **Samantha Perez‐Miller:** Methodology; validation; visualization; investigation. **Vlad Kumirov:** Methodology; validation; visualization; investigation; conceptualization. **Rajesh Khanna:** Methodology; validation; visualization; writing – original draft. **May Khanna:** Writing – original draft; conceptualization; investigation; funding acquisition; methodology; validation; visualization; writing – review and editing; supervision; project administration.

## Supporting information


**Data S1.** Supporting Information.

## Data Availability

The data that supports the findings of this study are available in the supplementary material of this article.
