# Supplementary material for: Identifying interactions between TDP‐43's N‐terminal and RNA‐binding domains
Source: Protein Sci. 2025 Sep 17;34(10):e70295. doi: 10.1002/pro.70295 (PMC12442443; doi:10.1002/pro.70295)
Supplement: Supplementary file 1 — Data S1. Supporting Information. [file PRO-34-e70295-s001.docx]

**Supplemental Figures**


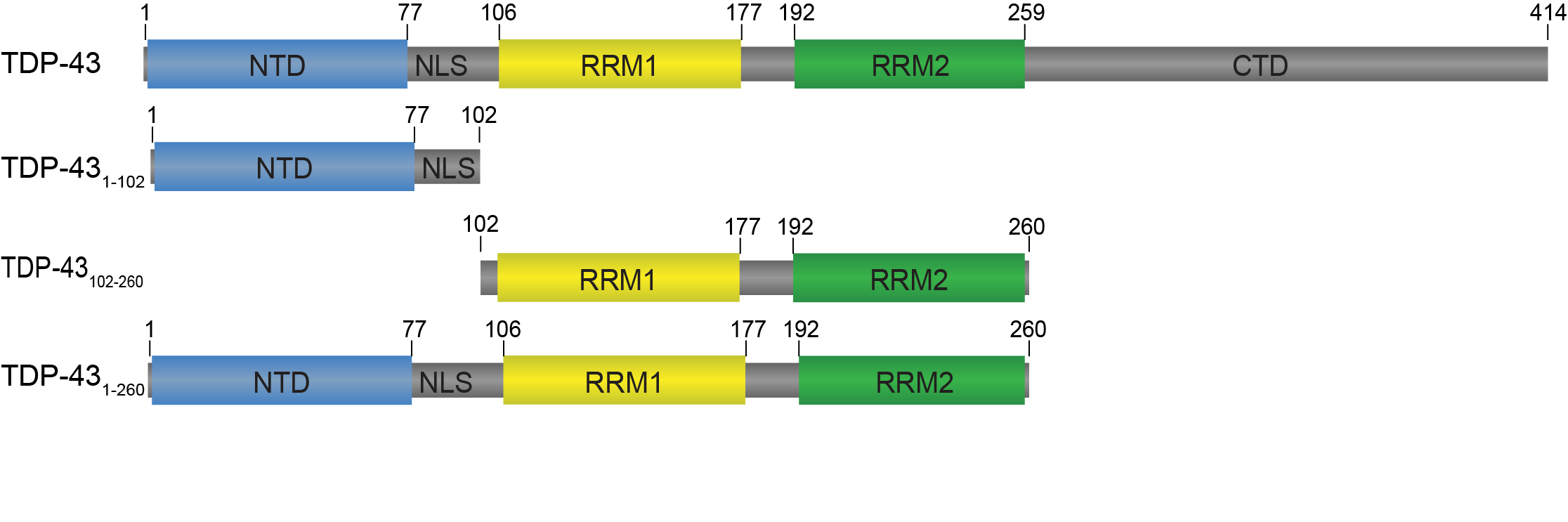


**Supplemental Figure 1. Domain architecture of subdomain constructs of TDP-43**


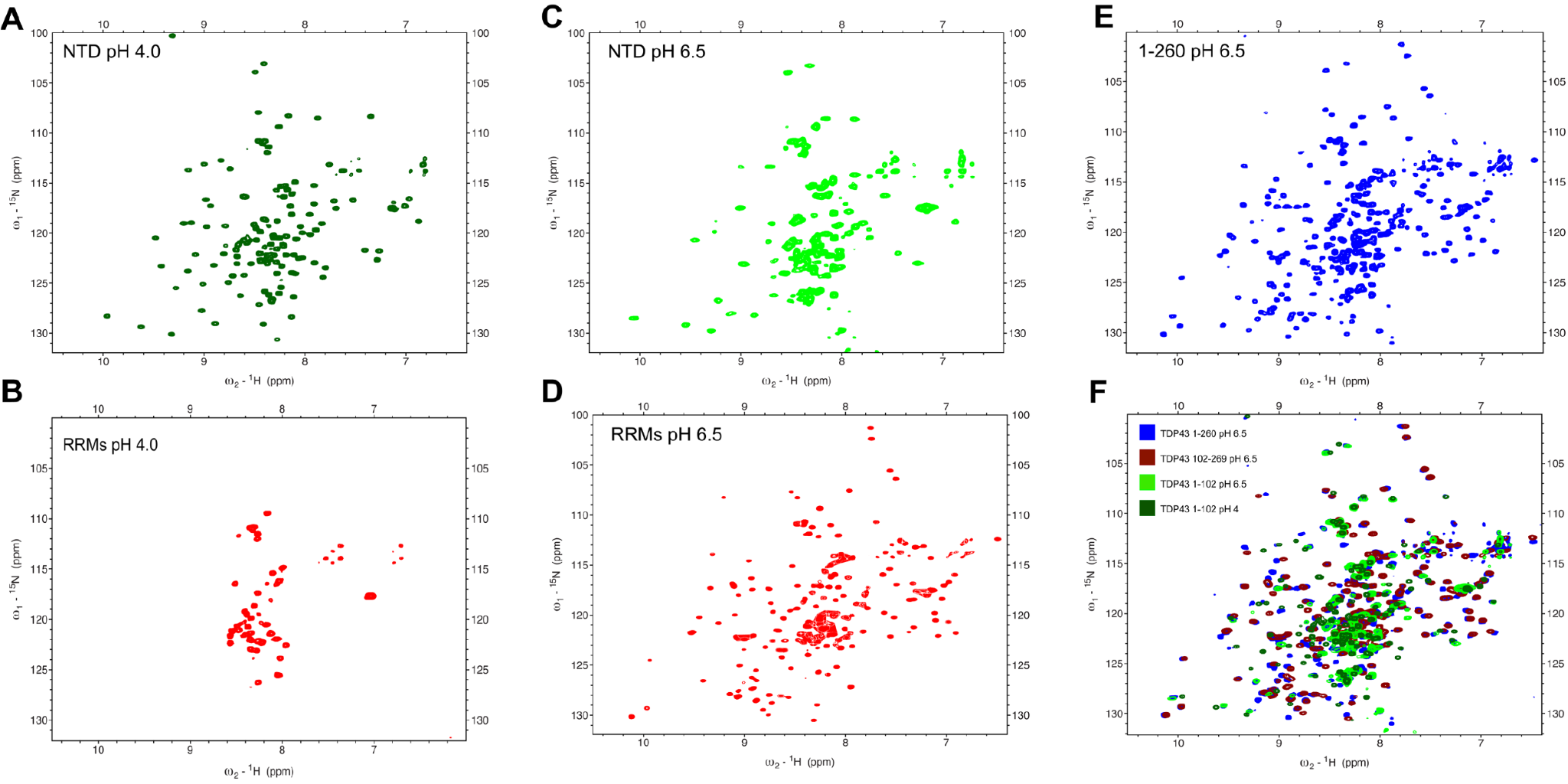


**Supplemental Figure 2: pH stability of TDP-43s NTD_1-102_, RRMs_102-260_, and a multidomain construct TDP-43_1-260_.** The pH stability of different constructs was tested. **A.** NTD (1-102aa) at pH 4.0 **B.** The RRM domain was tested at pH 4.0 (102-260 aa) **C.** NTD (1-102 aa) at pH 6.5 D. The RRM domain (1-260 aa) at pH 6.5 **E.** NTD-RRM (1-260 aa) domain at pH 6.5 and **F.** Overlay of the NTD-RRM (1-260 aa) at pH 6.5(blue) with TDP-43 RRM (102-269) at pH 6.5(burgundy) with TDP-43 NTD (1-102 aa) at pH 6.5 (green) with the NTD (1-102) at pH 4.0 (dark green).


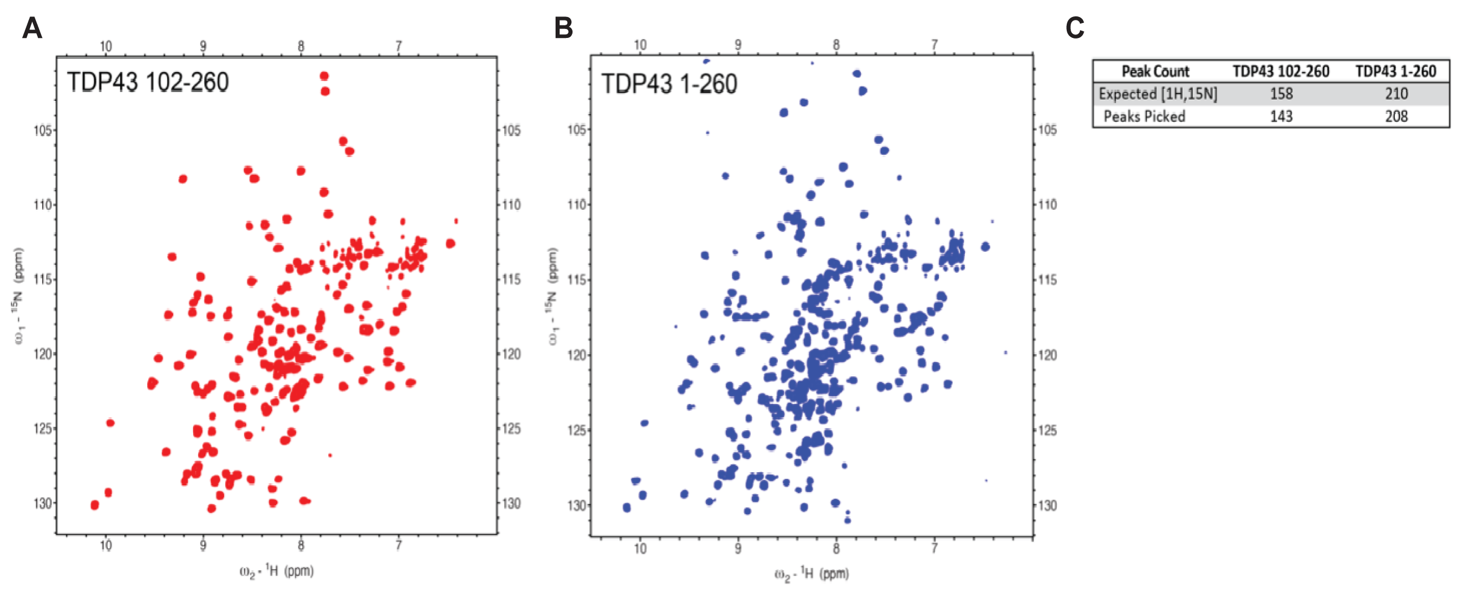


**Supplemental Figure 3. 2D [1H,15N] TROSY HSQC-NMR of TDP-43 subdomains**

**A.** TDP-43_102-260_ acquired at 25μM in NMR buffer shows a highly resolved spectrum with well-dispersed sharp peaks. **B.** TDP-43_1-260_ shows high resolution at 25μM resulting in well dispersed and sharp peaks for both the RRM domains and NTD **C.** Expected peak count of TDP-43 sequences is calculated from sequence length subtracted by the total number of proteins and linkers. Manual peak picking excluded side-chain resonances and identified ~95% of expected peaks for TDP-43.


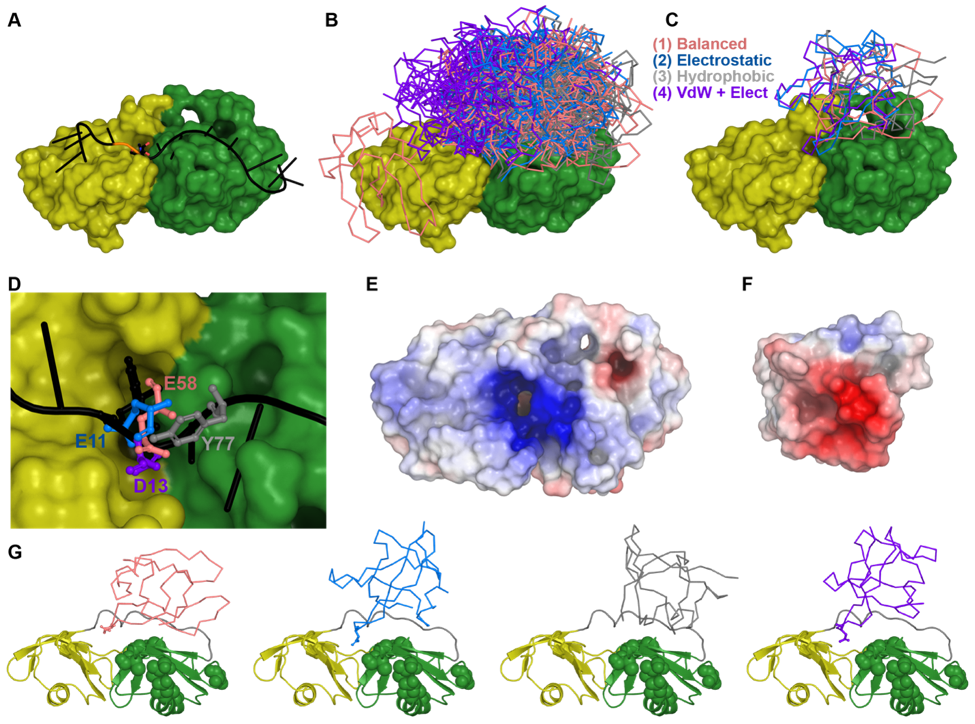


**Supplemental Figure 4.** **ClusPro docking of NTD to RRM1-RRM2.** **A**. NMR structure of RRM1 (yellow) and RRM2 (green) shown in surface representation with RNA as a cartoon (PDB ID 4BS2 ^2^). **B.** All models of docked NTD (PDB ID 2N4P ^29^) are shown as ribbons, colored by scoring function: (1) balanced models in pink, (2) electrostatic-favored models in blue, (3) hydrophobic-favored models in gray, and (4) van der Waals (VdW) + electrostatic favored models in purple. **C.** Top models for all four scoring methods. **D.** Location of residues overlapping with the RNA C’5 site. **E.** Electrostatic surface of RRM1-RRM2 and **F.** of NTD, where NTD was rotated 180 degrees about the vertical to show the surface that contacts RRM1-RRM2. **G.** Comparison of top model positions to chemical shift perturbations (green spheres).


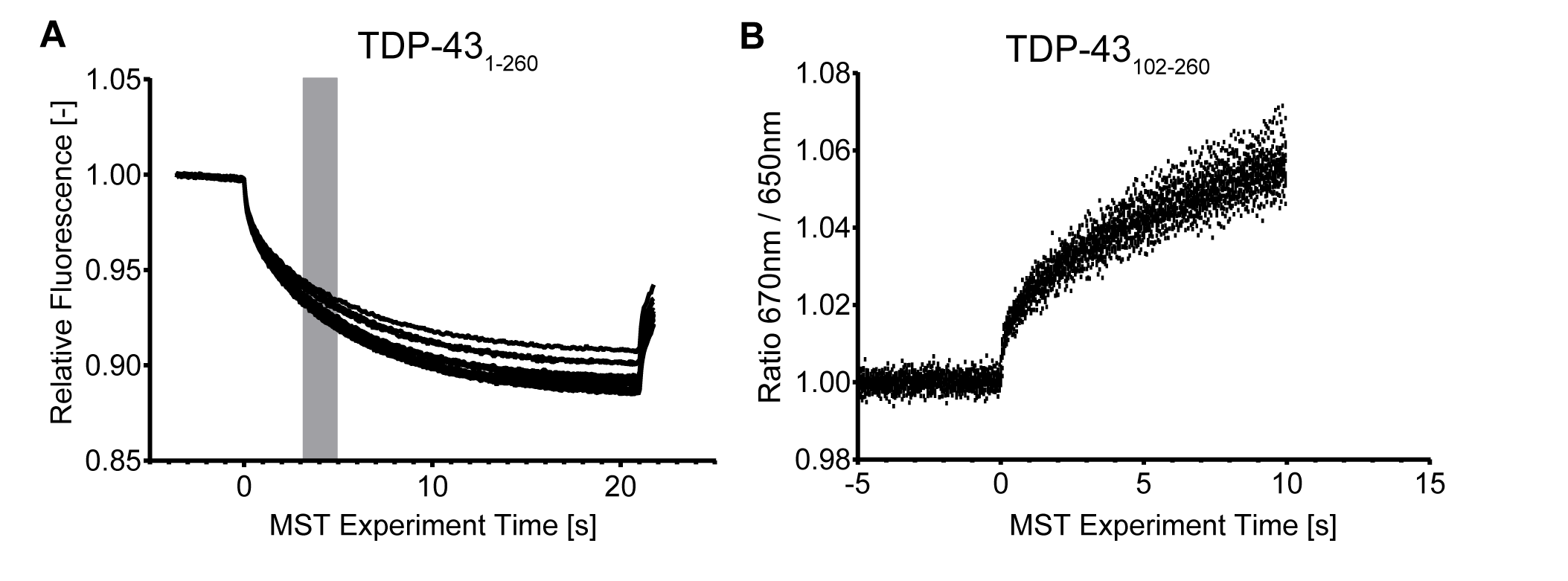


**Supplemental Figure 5. Representative MST Thermographs from data acquisition. A.** Representative set of thermographs of (UG)_4_ binding in increasing concentrations (0.24 nM to 4000 nM) to the NT-647 labeled HisTDP-43_1-260_ showed altered thermodiffusion, resulting in well-defined curves resembling a dose-response of the labeled-target when bound with a ligand. The grey bar (at ~5 s) indicates the steady-state time point used for analyzing the MST measurements in the graphs displayed in panel **B.** This representative set of thermographs shows the thermophoretic movement of (UG)_6_ when bound to NT-647 labeled HisTDP-43_102-260_ at increasing concentrations (0.006 nM to 2000 nM). The fluorescence signals were recorded at 650 nm and 670 nm, and their ratio plotted against the increasing concentrations of (UG)_6_ showed altered thermodiffusion, resulting in well-defined curves.


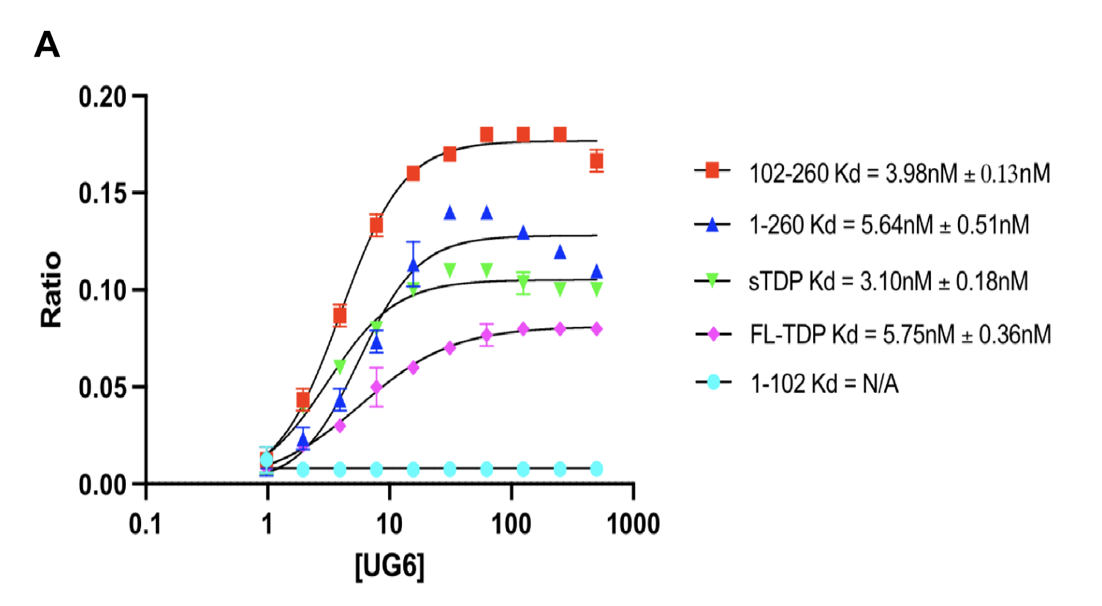


**Supplemental Figure 6. Homologous Time Resolved Fluorimetry (HTRF) of TDP-43 constructs and UG_6_. A.** The graph represents binding curves for five different complexes, each measured at varying concentrations of ligand (UG)_6_. The Y-axis shows the HTRF ratio of 620 nm and 665 nm, and the X-axis shows the ligand concentration on a logarithmic scale in the range of 0.1 nM to 1000 nM. The dissociation constant (Kd) values, indicated in the legend, demonstrate the binding affinity for each complex. As highlighted above, the NTD construct TDP-43_1-102_ shows no binding for RNA.


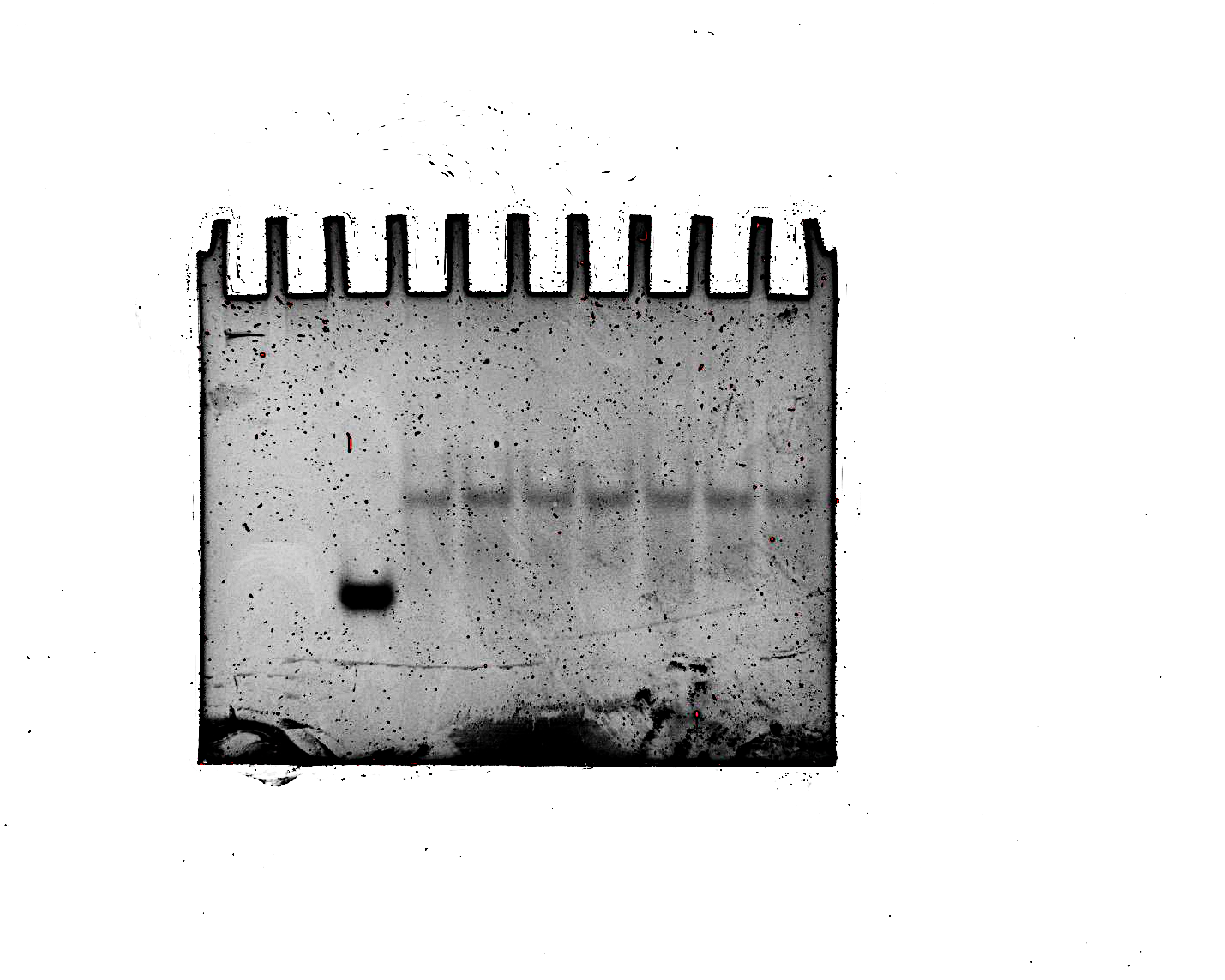


1 2 3 4 5 6 7 8 9

RNA

RNA:RRM

**Supplemental Figure 7. Native gel of RNA (250nM) bound to RRM (TDP-43_102-260_) with addition of NTD (TDP-43_1-77_).** Lane 1 is RRM alone (1uM) that shows no RNA bound. Lane 2 is 250 nM of (UG)_6_ RNA alone. Lanes 3 to 9 contain the same concentration of RNA along with 1 µM of RRM domain with the addition of increasing concentration of NTD: 0.1, 0.5, 1.0, 1.5, 2.0, 2.5, 5.0 µM.


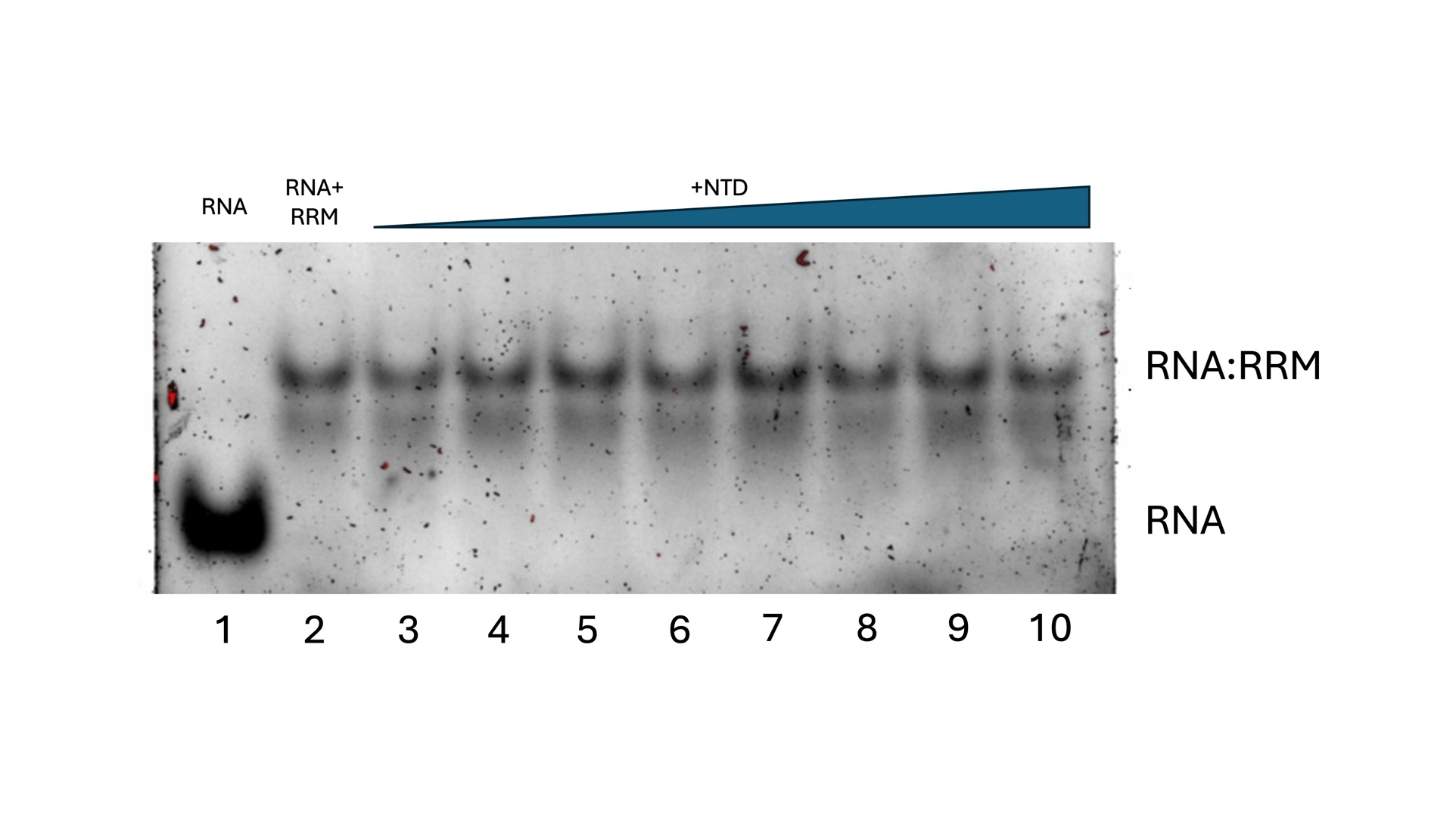


**Supplemental Figure 8. Native gel of RNA (500nM) bound to RRM (TDP-43_102-260_) with addition of NTD (TDP-43_1-77_).** Lane 1 is 500 nM of (UG)_6_ RNA alone. Lane 2 is 500 nM of (UG)_6_ RNA bound to 1 µM of RRM domain. Lanes 3–10 contain all the components of lane 2 along with increasing concentration of NTD: 0.1, 0.3, 1.0, 3.0, 5.0, 7.0, 10, 16.8 µM.


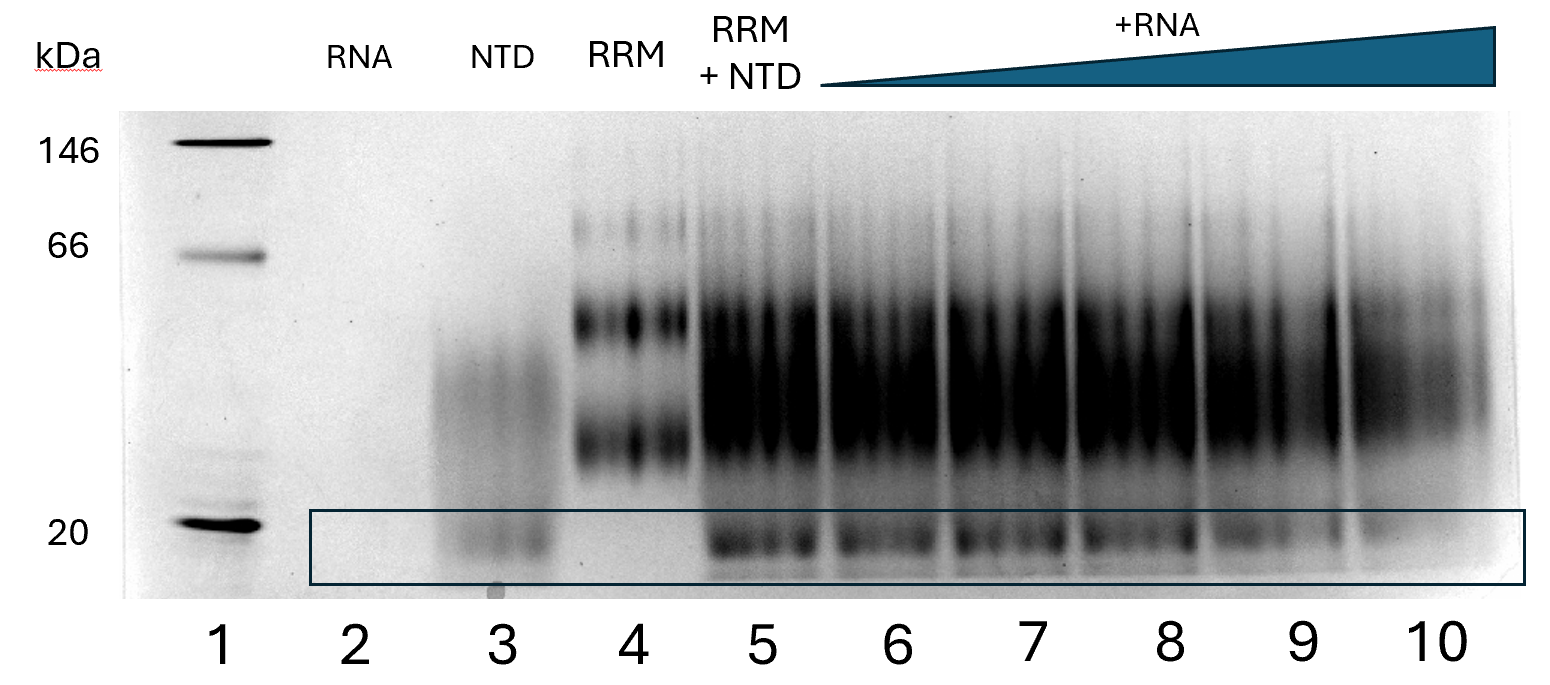


**Supplemental Figure 9. Native gel analysis of RRM (TDP-43_102-260_) interaction with NTD (TDP-43_1-77_) in the presence of RNA**. In order, lanes 2-4 contain 500 nM of (UG)_6_ RNA alone, 16.25 µM of NTD alone, and 8.125 µM of RRM alone. Lane 5 contains 8.125 µM RRM and 16.25 µM NTD, showing complex formation. Lanes 6-10 contain all the components of lane 4 with increasing concentration of (UG)_6_ RNA: 10, 33, 100, 250, and 500 nM.
